# Nickel Sensitivity Is Associated with GH-IGF1 Axis Impairment and Pituitary Abnormalities on MRI in Overweight and Obese Subjects

**DOI:** 10.3390/ijms21249733

**Published:** 2020-12-20

**Authors:** Renata Risi, Simonetta Masieri, Eleonora Poggiogalle, Mikiko Watanabe, Alessandra Caputi, Rossella Tozzi, Elena Gangitano, Davide Masi, Stefania Mariani, Lucio Gnessi, Carla Lubrano

**Affiliations:** 1 Department of Experimental Medicine, Section of Medical Pathophysiology, Food Science and Endocrinology, Sapienza University of Rome, Viale Regina Elena, 324, 00161 Rome, Italy; renata.risi@uniroma1.it (R.R.); Eleonora.poggiogalle@uniroma1.it (E.P.); mikiko.watanabe@uniroma1.it (M.W.); Alessandra.caputi@uniroma1.it (A.C.); rossella.tozzi@uniroma1.it (R.T.); elena.gangitano@uniroma1.it (E.G.); davide.masi@uniroma1.it (D.M.); s.mariani@uniroma1.it (S.M.); lucio.gnessi@uniroma1.it (L.G.); 2 Department of Sensory Organs, Sapienza University of Rome, 00161 Rome, Italy; simonetta.masieri@uniroma1.it

**Keywords:** obesity, nickel sensitivity, growth hormone deficiency, pituitary morphology

## Abstract

Nickel (Ni) is a ubiquitous metal, the exposure of which is implied in the development of contact dermatitis (nickel allergic contact dermatitis (Ni-ACD)) and Systemic Ni Allergy Syndrome (SNAS), very common among overweight/obese patients. Preclinical studies have linked Ni exposure to abnormal production/release of Growth Hormone (GH), and we previously found an association between Ni-ACD/SNAS and GH-Insulin-like growth factor 1 (IGF1) axis dysregulation in obese individuals, altogether suggesting a role for this metal as a pituitary disruptor. We herein aimed to directly evaluate the pituitary gland in overweight/obese patients with signs/symptoms suggestive of Ni allergy, exploring the link with GH secretion; 859 subjects with overweight/obesity and suspected of Ni allergy underwent Ni patch tests. Among these, 106 were also suspected of GH deficiency (GHD) and underwent dynamic testing as well as magnetic resonance imaging for routine follow up of benign diseases or following GHD diagnosis. We report that subjects with Ni allergies show a greater GH-IGF1 axis impairment, a higher prevalence of Empty Sella (ES), a reduced pituitary volume and a higher normalized T2 pituitary intensity compared to nonallergic ones. We hypothesize that Ni may be detrimental to the pituitary gland, through increased inflammation, thus contributing to GH-IGF1 axis dysregulation.

## 1. Introduction

Obesity is a growing health problem that has reached epidemic proportions and has led to a skyrocketing increase in the prevalence of many of its complications, some of which are well established, namely diabetes mellitus type 2, cardiovascular disease, obstructive sleep apnoea syndrome (OSAS), non-alcoholic fatty liver disease (NAFLD) and sarcopenia [1,2,3,4,5], and others are currently being further investigated [6,7]. It has been predicted that, by 2025, the global obesity prevalence will reach 18% in men and 21% in women [8], whereas in some areas, such as southern and insular regions of Italy, the obesity rate has already been reported as high as 32% [9]. As the prevalence of obesity is steadily increasing, much attention has been paid both to the possible treatments [10,11,12,13,14,15,16,17,18,19] and to the identification of additional/alternative pathogenetic pathways [20], such as the environmental factors involved in obesity development [21].

Nickel (Ni) is a highly allergenic metal, widely used in a large variety of products and ubiquitous in the environment. Common sources of Ni are costume jewellery, coins, mobile phones and dental materials [22], but it could also be ingested, being naturally found in drinking water and in various food, such as chocolate, legumes, shellfish, grains, nuts and canned products [23]. Studies have shown that Ni exposure cause several health problems such as liver, kidney, spleen, brain and other tissues damage; vesicular eczema; and lung and nasal cancer [24]. Moreover, occupational exposure to Ni and its compounds in sensitized individuals can also cause allergic dermatitis known as nickel allergic contact dermatitis (Ni-ACD), the most common cutaneous delayed-type hypersensitivity (type IV) reaction worldwide; Ni-ACD prevalence in the general population is 8–18% in the US and Europe, and it is estimated to reach 16% in southern European countries such as Italy [25,26]; however, Ni allergy may also manifest as Systemic Ni Allergy Syndrome (SNAS), a condition occurring in a considerable number of patients sensitized to Ni and characterized by cutaneous and extracutaneous signs and symptoms [27]. Ni-ACD/SNAS prevalence decreased in countries that followed the 1994 EU directive regulating the amount of Ni in commonly used products, such as Denmark and Sweden, proving that Ni allergy is largely attributable to its exposure [25]. Of note, Southern European countries did not abide, and they report higher prevalence data (16% compared to 10% of northern countries). [25]. In particular, it has been demonstrated that increased Ni levels in ambient air are associated with higher urinary Ni concentrations and a greater prevalence of Ni sensitization in children [28]. Moreover, the European Food Safety Authority (EFSA) recently released an alert stating that exposure to current levels of Ni in food and water represents a risk factor for the development of health conditions in individuals sensitized to Ni [29]. Noteworthily, reducing dietary Ni intake ameliorates SNAS symptoms [30,31]. Ni, whether taken orally or parenterally, accumulates in the body [32,33,34]. In particular, the body burden of Ni in adult humans averages about 0.5 mg per 70 kg, with the highest concentrations found in the lungs, in the thyroid and in the adrenal glands (about 20–25 μg/kg wet weight) [34], although traces of the metal have also been detected in the kidney, liver and brain [34] and in human adipose tissue [33]. Evidence suggests that Ni allergy/exposure may have a role in the complex mechanisms underlying obesity pathogenesis. First, Ni allergy is much more common in patients with obesity compared to the general population [35], and it is even more widespread among obese patients with worse metabolic profiles [36]; moreover, obese patients sensitized to Ni have a significantly lower baseline insulin-like growth factor 1 (IGF-1) level and a blunted growth hormone (GH) dynamic response compared to nonallergic ones [36]. The contribution of Ni allergy to fat mass excess could be immune-mediated, since both SNAS [37] and obesity [38] share an enhanced systemic inflammatory response as a fundamental pathogenetic mechanism; furthermore, it is known that exposure to different metals, such as Ni, can act as a trigger of autoimmune disorders, eventually leading to neurotoxicity [39]. Emerging evidence also indicates a direct role of Ni in altering specific metabolic pathways, and preclinical studies have recently shown that Ni may influence energy metabolism and glucose homeostasis [40,41,42,43,44,45]. Moreover, studies on animals and human subjects have prospected that Ni could exhibit endocrine-disrupting activity, being able to alter normal synthesis and/or secretion of GH [46,47,48,49]. In this scenario, it is worth recalling that fat mass excess is associated with profound structural changes of neurons and glia in the hypothalamus [50] and with the presence of Empty Sella (ES), a complex condition which may eventually lead to hypopituitarism and, in particular, to adult-onset GH deficiency (GHD) [51]. The relationship between GHD and obesity is notoriously ambivalent, and the question of which pathogenetic event occurs first is still a matter of debate; in fact, fat mass excess usually results in a blunted GH response, that in turn contributes to weight gain [52]. It has been reported that Magnetic Resonance Imaging (MRI) performed on the pituitary is able to reflect specific endocrinological alterations: for example, GHD seems to be linked to pituitary height and volume in paediatric patients [53,54,55] and to ES in obese adults [56]. Interestingly, excessive exposure to some metals might result in their brain deposition, often leading to specific MRI findings and functional abnormalities [57]; in particular, iron deposition within the pituitary in thalassaemic patients is typically associated with mean signal intensity alterations on MRI as well as GH-IGF1 axis impairment and hypogonadism [58,59]. However, next to nothing is known about the possible harmful effects of Ni on the human pituitary.

Given the high prevalence of both Ni allergy and obesity in the general population and the hints coming from studies supporting a possible correlation between Ni allergy/exposure and GH-IGF1 axis disruption, we herein aimed to evaluate the relation between pituitary morphology and GH secretion in a cohort of overweight and obese subjects who were subjected to a Ni patch test for symptoms and signs suggestive of Ni-ACD/SNAS.

## 2. Results

### 2.1. Demographic, Anthropometric and Metabolic Parameters

Accessing the High Specialization Center for the Care of Obesity, Sapienza University of Rome, from 2010 to 2018, 1526 subjects were evaluated; 70% (*n* = 1081) reported complaints compatible with ACD/SNAS. Of these, 79.4% (*n* = 859) met inclusion criteria and exclusion criteria and were therefore subjected to a 5% Ni sulphate patch test. Among the patients undergoing the diagnostic procedure, 106 underwent pituitary MRI as part of their diagnostic route and 67 met the inclusion criteria and were included in the study. Of these, 62.7% (*n* = 42) were positive to a Ni patch test and 37.3% (*n* = 25) were negative. The demographic, anthropometric and metabolic characteristics of the patients are shown in Table 1. The population was similar in gender distribution and BMI. Half of the patients with Ni allergy were diagnosed with metabolic syndrome, according to the National Cholesterol Education Program (NCEP) Adult Treatment Panel III (ATP III) criteria [60]. The percentage of patients with impaired glucose and lipid metabolism and with hypertension was higher in the Ni-sensitive group (Table 1).

### 2.2. Pituitary Homonal Status

Pituitary hormones differences between the Ni allergic and nonallergic subjects are reported in Table 2. Ni allergy was associated with reduced serum levels of IGF-1 and 8 AM serum cortisol, and with higher adrenocorticotropic hormone (ACTH) plasma concentrations. Of note, in all patients, both ACTH and cortisol serum levels were in the normal ranges.

**Figure 1:**
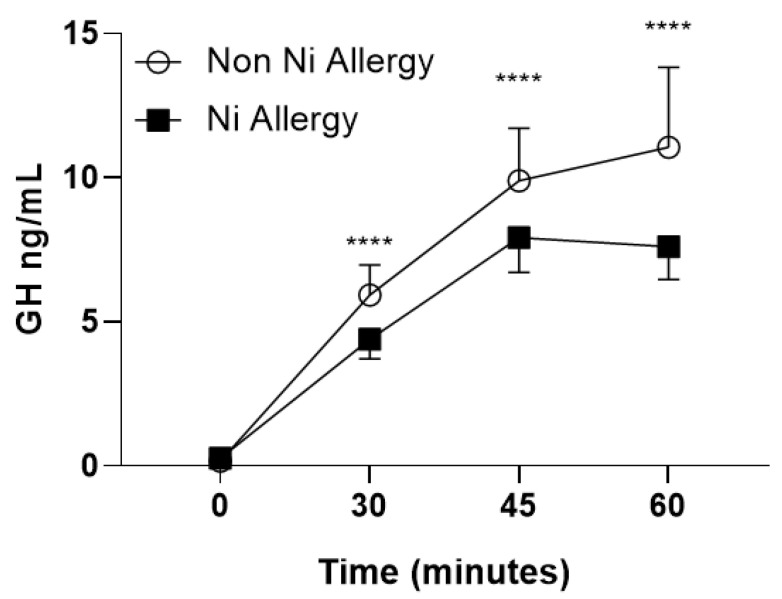
GHRH + arginine dynamic test: in non-Ni allergy patients, GH level significantly rose at 30′, 45′ and 60′ from basal level (*p* < 0.0001, ****) while Ni allergic patients showed a blunted response, with nonsignificant increase in GH level during the test. Abbreviation: Ni, Nickel; GH, Growth Hormone; GHRH, growth hormone releasing hormone. **** *p* < 0.0001.

Ni allergic patients also showed a blunted GH dynamic response upon GHRH + arginine stimulus compared to nonallergic ones (Figure 1).

GHD was diagnosed in 4 patients out of 25 (16%) who were proven to be nonallergic and in 13 patients out of 42 (30.9%) diagnosed with Ni allergy. The 4 nonallergic patients with GHD as well as 12 out of 13 of the subjects sensitized to Ni were all female.

### 2.3. Pituitary Morphology on MRI

In our population, 19 patients out of 42 (45.2%) sensitized to Ni showed MRI-derived pituitary morphology compatible with ES, with a mean age of 52.8 ± 6.3 years old. Of these, 18 out of 19 were female (94.7%). Conversely, only 7 patients out of 25 nonallergic subjects (28%) received the same diagnosis; they were younger (35.6 ± 17.9 years old) than the Ni allergy group. Female gender was still predominant, with 6 out of 7 subjects being women. Ni allergy was overall associated with significant lower pituitary height and indirectly estimated PV, while pituitary width and length, and pituitary stalk thickness were not different between the groups (Table 3).

After excluding subjects with radiological evidence of ES, PV was still significantly lower in the Ni allergy group compared to the non-Ni allergy one, whereas pituitary diameters and stalk thickness failed to reach a statistically significant difference (Table 2). Intriguingly, T2-weighted pituitary to muscular mean signal intensity (T2W PTM intensity) was higher in Ni allergic subjects compared to nonallergic ones, whereas T1-weighted pituitary to muscular mean signal intensity (12W PTM intensity) was not different between the groups (Table 2).

### 2.4. Bivariate and Partial Correlations between Ni Allergy, BMI, GH-IGF1 Axis and Inflammatory Markers in the Study Population

Bivariate and partial correlations between Ni allergy, BMI, GH-IGF1 axis and inflammatory markers are summarized in Table 4 and Table 5. Briefly, Ni allergy was negatively associated with baseline IGF-1 serum levels and pituitary volume and positively associated with T2W pituitary to muscular intensity (Table 4). After adjustment for BMI, Ni allergy maintained its inverse correlation with PV (Table 5) and became positively correlated with the inflammatory markers evaluated (total number of white blood cells, lymphocytes, neutrophils and C-reactive protein) (Table 5). Baseline IGF-1 serum levels positively correlated with the number of white blood cells (WBC) and negatively correlated with the number of lymphocytes (Table 4). However, after adjustment for BMI, these correlations were lost (Table 5). Finally, the GH peak upon GHRH + arginine test showed an inverse correlation with C-Reactive Protein (CRP) after BMI adjustment (Table 5).

## 3. Discussion

Ni-ACD and SNAS are common and complex conditions for which pathogenesis is yet to be fully elucidated, although Ni exposure is known to play a relevant role [25,28]. It has been reported that exposure to current levels of Ni in food and water may be dangerous in sensitized subjects [29], and this is alarming considering that Ni accumulates in several organs [32,33,34]. Ni-ACD and SNAS prevalence is higher in people with obesity compared to the general population, and in these subjects, the diagnosis of Ni allergy is associated with worse metabolic profile and impaired GH-IGF1 axis [36]. The relationship between Ni allergy and metabolic disruption is still unclear, although the immune system could be a possible link [37,38]; moreover, evidence from recent preclinical studies suggest that Ni exposure influences energy metabolism and glucose homeostasis [40,41,42,43,44,45] and that it is able to alter the normal production/release of GH [46,47,48,49]. In the current study, we evaluated the pituitary morphology of obese and overweight patients undergoing Ni patch tests because of suspected Ni allergy and we focused on the link with GH secretion. We confirmed that allergic individuals had worse metabolic status, lower baseline IGF-1 levels and blunted GH response upon dynamic testing compared to the nonallergic counterpart, who had similar age, BMI, and waist and hip circumference. We also observed that the prevalence of GHD was slightly higher among Ni allergic subjects compared to nonallergic ones, and the presence of Ni allergy was inversely correlated with baseline IGF1 levels. More importantly, for the first time, we found that Ni allergy correlates not only to functional but also to morphological abnormalities. First, we observed a higher prevalence of ES in subjects sensitized to Ni compared to nonallergic ones, reaching 45.2% in the Ni allergy group, that is significantly higher than the prevalence of ES reported in the general population, where it ranges from 5.5 to 35% [51]. We also found that the pituitary volume was reduced in obese/overweight patients with Ni allergy compared to the nonallergic counterpart, and this is mainly attributable to the reduction in pituitary height, with the gland length and width being similar between the two groups. Intriguingly, even after eliminating the subgroup of patients with MRI findings compatible with ES, as they may potentially represent a bias, Ni allergy was still correlated to smaller pituitary size, suggesting that this association is independent of the presence/absence of ES. The negative association between the presence of Ni allergy and the pituitary volume was maintained after BMI adjustment. Our findings substantially confirm in adults what was previously observed in paediatric patients: that GHD is associated with lower pituitary size [53,54,55], and with the presence of ES [56]. In addition, they add a piece to the understanding of the complex mechanisms underlying Ni allergy and metabolic disruption, namely that the hormonal impairment observed in Ni-sensitized patients is mirrored by organic pituitary damage; the evidence of concomitant GH-IGF1 axis impairment and reduced pituitary volume in Ni-sensitized subjects strengthens this hypothesis. Interestingly, we also found that T2 pituitary mean signal intensity normalized by the muscular one was higher in the group of patients sensitized to Ni compared to the nonallergic counterpart; this observation may exclude that the hypothesized Ni-induced pituitary damage could be derived from metal accumulation such as in the case of iron overload [58,59]; in fact, with Ni being a ferromagnetic metal as well as iron, contrary to our findings, its abnormal brain deposition should lead to a decrease in the pituitary intensity in T2 scans [57]. We could speculate that Ni exposure/allergy may lead to pituitary damage of an inflammatory nature. In fact, it is known that pituitary size reduction often represents the consequence of various types of hypophysitis [61] and that there is a strong association between SNAS and the increased systemic inflammation [37,39], also confirmed in our study population, where the presence of Ni allergy was positively correlated with several inflammatory markers. Of note, nonallergic subjects with radiological finding of ES were younger than the allergic counterpart (35.6 ± 17.9 years old compared to 52.8 ± 6.3 years old, respectively), suggesting that Ni exposure/allergy may lead to pituitary damage only in the long term. The evidence that Ni is a direct activator of toll-like receptor 4, which in turn triggers an immunological response [62], and the observation that the same receptor is expressed in the pituitary [63,64] may further reinforce this hypothesis. In this context, the finding of ES, that we found more frequently in Ni allergy patients, might be the final stage of progressive pituitary volume reduction following inflammatory injury, as observed in mice with autoimmune hypophysitis [65]. Accordingly, it could be speculated that the increase in T2 pituitary signal intensity we found may reflect an inflammatory infiltrate, although this hypothesis should be reconfirmed with histological ad hoc studies. Of note, T2 pituitary signal intensity was positively correlated with the presence of Ni allergy, but it did not show any correlation with the inflammatory status in our study. Our study has some limitations that should be acknowledged. First, given the retrospective study design, some important data, such as ES-related clinical symptoms, were not collected, although they were not spontaneously reported by any patient following accurate medical history collection. Second, its observational nature prevents us from drawing any conclusion on the cause–effect relationships occurring between Ni allergy, obesity, and GH-IGF1 axis and pituitary morphological abnormalities. In fact, since each of these variables appear reciprocally linked with the other, it would be hardly impossible to state which is the first pathogenetic event among them. However, given the potential role of Ni as an endocrine disruptor observed in preclinical studies [46,47,48,49], it appears reasonable to assume that the association between Ni allergy and metabolic disruption may not be merely epidemiological and that the metal might exert direct or indirect detrimental effects at the pituitary level, ultimately resulting in the observed morphological and hormonal impairment. Third, the lack of a control group composed by normal-weight patients, in which diagnosis of GHD is less likely, prevents us from evaluating the impact of Ni exposure on nonobese subjects. Fourth, it was not possible in our clinical setting to directly assess Ni exposure through the measurement of serum or urinary Ni concentrations; however, evidence in the literature suggests that Ni ACD/SNAS prevalence is largely attributable to Ni exposure [25,28], and we therefore considered Ni allergy prevalence as an indirect marker of metal exposure. Fifth, we compared MRI findings of different scans, and despite the standardization of pituitary intensity that we adopted that should have overcome this limit, data related to signal intensities should be reconfirmed with targeted radiological studies. Sixth, considering that ES may represent a developmental defect, it would have been worthy to have information regarding Ni exposure/allergy in pregnant mothers. However, given the retrospective design of the study, we could not collect these data. Seventh, the finding of lower serum levels of morning ACTH and cortisol, although still in the normal ranges, in patients sensitized to Ni should be reconfirmed and further investigated in a proper study. In fact, we excluded a priori other pituitary axis impairments in order to include a highly selected population to fit our primary aim, which was the evaluation of the link between GH/IGF1 axis, pituitary morphology and Ni allergy. However, we cannot exclude that Ni exposure may influence other pituitary axes as well. Finally, the sample size was relatively small, and it was not homogenous gender-wise, with a predominance of female sex. The discrepancy in gender distribution is explained by the fact that the population accessing our centre is composed mostly by female subjects, who more frequently consider obesity as a disease and therefore seek medical assistance for it [66]. Moreover, several signs and symptoms possibly pointing at ACD or SNAS such as bloating, fatigue and headache are more frequently reported by female individuals [67]. Further studies with a larger study population comprising both men and women in equal proportions are required in order to validate our findings. Despite the limitations, this is the first study evaluating the GH-IGF1 axis and the pituitary morphology in subjects with Ni allergy. If our generated hypotheses were validated, Ni exposure would become an urgent public health issue, considering the metal ubiquity in our environment and the epidemic spread of obesity worldwide. Moreover, it would also shed light on a possible therapeutic strategy to deal with obesity, especially when associated with Ni allergy: in fact, a low Ni diet has been demonstrated not only to ameliorate systemic Ni allergy symptoms [28,29] but also to induce weight loss in patients allergic to Ni [30]. However, many questions remain to be answered; thus, further studies evaluating Ni metabolism and deposition in specific sites of human body as well as the direct and indirect effects of Ni exposure in vivo in target organs such as the pituitary gland are needed to fully complete the picture.

## 4. Patients and Methods

### 4.1. Subjects

**Figure 2:**
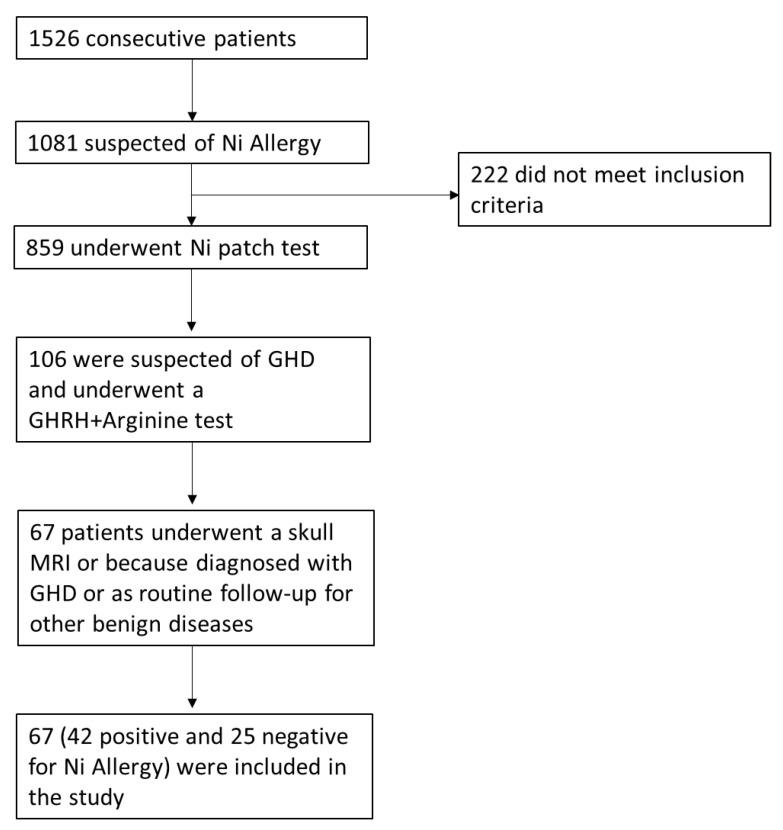
Flowchart of patient enrolment. Abbreviations: Ni, Nickel; GHD, Growth Hormone Deficiency; GHRH, Growth Hormone Releasing Hormone; MRI, Magnetic Resonance Imaging.

Accessing the High Specialization Center for the Care of Obesity, Sapienza University of Rome, from 2010 to 2018, 1526 subjects were clinically evaluated as previously reported in the study of Watanabe et al., which was partially conducted on the same cohort of patients [36]. A flowchart of patient enrolment is represented in Figure 2.

All patients had their medical history collected, physical exam and laboratory work performed as part of routine diagnostic workup during hospitalization. Whenever clinically required, dynamic tests and imaging studies were carried out. Patients presenting at least two signs or symptoms compatible with Ni-ACD/SNAS such as gastrointestinal distress (meteorism, bloating, abdominal pain, dyspepsia, diarrhoea and constipation), systemic symptoms (fatigue and headache) and cutaneous signs (itching, rash, dermographia and urticaria) were candidate to a Ni patch test [68]; exclusion criteria to undergo the Ni patch test were age < 18 and >65, systemic and topic treatment with corticosteroids, antihistamine or other immunosuppressive agents, pregnancy and absence of written informed consent. Among those subjected to the patch test, those showing IGF-1 levels (<50 percentile for age and sex) together with signs/symptoms of GHD were tested through Growth Hormone Releasing Hormone (GHRH) plus arginine infusion [61,69,70]. Of these, 67 patients underwent a brain MRI for routine follow-up of benign diseases (i.e., persistent headache, cerebral arteriovenous malformations and non-functional pituitary microadenoma) or following GHD diagnosis and were included in the present study.

Exclusion criteria were hypothalamic-pituitary-adrenal axis impairment, hypogonadism, central hypothyroidism, pituitary macroadenoma, any malignant cerebral disease, and previous treatment with cranial irradiation or surgery. All procedures performed were in accordance with the ethical standards of the institutional and/or national research committee and with the 1964 Helsinki declaration and its later amendments or comparable ethical standards. The study was reviewed and approved on the 24th of October 2019 by Sapienza University of Rome Ethics Committee.

### 4.2. Anthropometric Measurements

Anthropometric parameters were obtained between 8 and 10 a.m. in fasting subjects wearing light clothing and no shoes. Body weight were obtained with the use of a balance-beam scale (Seca GmbH & Co, Hamburg, Germany). Height was rounded to the closest 0.5 cm. Body Mass Index (BMI) was calculated as weight divided by squared height in meters (kg/m^2^). Waist circumference was measured at the iliac crest, and hip circumference was measured at the symphysis-greater trochanter level to the closest 1.0 cm.

### 4.3. Laboratory Assessments

Blood samples were collected from fasting patients by venipuncture between 8 and 9 a.m. Samples were then transferred to the local laboratory and handled according to the local standards of practice. Insulin-like growth factor 1 (IGF-1), growth hormone (GH), and routine laboratory tests, including metabolic and inflammatory markers, were measured. Specifically, IGF-1 and GH were assayed by radioimmuno-assay (Diagnostic System Laboratories Inc., Webster, TX, USA, and CISbio, Cedex, Saclay, France; DiaSorin S.p.A., Saluggia, VC, Italy, respectively).

### 4.4. Dynamic Test

Patients suspected of GHD were tested according to theAmerican Association of Clinical Endocrinology (AACE) guidelines with GHRH (GHRH 1–29; Ferring, Italy; 1 μg/kg BW iv at time 0) plus arginine (0.5 g/kg BW, L-ARG mono-hydrochloride by i.v., from time 0 over 30 min) [61]. Written informed consent for the medical procedure was obtained from all patients. Blood samples were drawn from an indwelling catheter inserted in an antecubital vein at times 0, 30, 45 and 60 min. All studies started between 8 and 9 AM, after overnight fasting. GHD was diagnosed according to AACE guidelines as a peak value below 4.8 ng/dL [61].

### 4.5. Patch Test

Patients with a clinical suspicion of Ni-ACD/SNAS meeting inclusion and exclusion criteria were subjected to Ni patch tests with the Società Italiana Dermatologia Allergologica Professionale ed Ambientale (SIDAPA) baseline series (Lofarma S.p.A., Milano, Italy) that assess Ni sensitivity with a 5% nickel sulphate solution. Finn Chambers^®^ (diameter, 8 mm; SmartPractice^®^, Phoenix, AZ, USA) on Scanpor^®^ tape (Norgesplaster A/S, Vennesla, Norway) were applied and left on the back for 48 h [68]. The readings were done on day 3 by a trained allergist. For patch test analysis, reactions “+” to “+++” were classified as positive and negative, and doubtful reactions were classified as non-positive.

### 4.6. Magnetic Resonance Imaging (MRI)

When pituitary MRI was performed at our hospital, a 1.5 T scanner (Signa HDx, General Electric, Milwaukee, WI, USA) with gadolinium contrast enhancement was used. We collected and included in the study also pituitary MRI performed at other institutions as well. The midsagittal and coronal T2 images centred at the pituitary stalk were used to measure the central height, width, length and cross-sectional area of the gland. All measurements were obtained independently with the aid of RadiAnt DICOM 2.2.9 Viewer (Medixant, Poznan, Poland) by two experienced physicians blinded to the clinical diagnosis. Pituitary volume was indirectly derived from the following formula as reported in previous studies [71,72]:

Pituitary volume (PV) = ½ × Length × Width × Height(1)

Standard criteria (cerebrospinal fluid occupying more than 50% of *sella turcica* (ST) with a compressed pituitary gland against the sellar wall) were adopted for MRI diagnosis of ES.

Pituitary mean signal intensity was taken from the mid coronal T2 and T1-weighted images centred at the pituitary stalk. The mean signal intensity of muscular tissue was calculated from the same image by averaging the mean signal intensities of 4 spheroidal region of interest (ROI) of 10 mm in diameter placed at the level of the medial and lateral pterygoid muscles bilaterally. In order to standardize the pituitary intensities of different scans, the pituitary mean signal intensity was normalized to the muscular mean signal intensity and the following parameters were calculated:

T1-weighted Pituitary to Muscular mean signal intensity (T1W PTM intensity): T1-weighted pituitary mean signal intensity/T1-weighted muscular mean signal intensity;

T2-weighted Pituitary to Muscular mean signal intensity (T2W PTM intensity): T2-weighted pituitary mean signal intensity/T2-weighted muscular mean signal intensity.

### 4.7. Statistical Analysis

Normality was assessed with the Shapiro–Wilk test. Variables were normally distributed and expressed as mean ± standard deviation (SD). Independent samples Student’s *t*-test and Chi-squared test were used to assess differences between groups as appropriate. Differences between GH levels among groups upon dynamic testing were assessed by repeated measures analysis of variance (ANOVA). Bivariate and partial correlations between variables under evaluation were performed. Differences were considered statistically significant when *p* < 0.05. Statistical analysis was performed using GraphPad Prism Version 8.00 for Windows, GraphPad Software, San Diego California USA and SPSS Statistics for Windows, Version 25.0, Armonk, NY, USA: IBM Corp.

## 5. Conclusions

In conclusion, our work demonstrates that overweight/obese patients with Ni allergy show worse metabolic status, GH-IGF1 axis impairment, higher prevalence of ES and reduced pituitary volume compared to nonallergic ones. We hypothesized that Ni may be detrimental to the pituitary gland, leading to hormonal and structural abnormalities, which could in turn contribute to obesity pathogenesis and metabolic disruption. We also speculate that the nature of Ni-induced pituitary damage may be inflammatory. However, the study design does not allow us to draw conclusions regarding the cause–effect relationships underlying these pathogenetic events, and further works are required to investigate to what extent Ni exposure/allergy impacts human health.

## Figures and Tables

**Table 1:** General and metabolic differences between patients testing positive or negative to a Ni patch test are shown. Abbreviations: SD, Standard Deviation; Ni, Nickel; BMI, body mass index; Met-S, Metabolic Syndrome; IFG, impaired fasting glucose; DM, diabetes mellitus; HT, hypertension. * *p* < 0.05. ** *p* < 0.01.

Demographic, Anthropometric and Metabolic Parameters	Ni Allergy Confirmed (*n* = 42)	Ni Allergy Excluded (*n* = 25)
	Mean ± SD	Mean ± SD
Age (years)	48.1 ± 1.6	43.3 ± 1.7
Gender (%F)	95.9	84.6
Weight (Kg)	94.2 ± 22	97.7 ± 20.5
BMI (Kg/m^2^)	34.9 ± 6.1	35.1 ± 6.6
Waist circumference (cm)	112.6 ± 17.3	114 ± 15.2
Hip circumference (cm)	118.3 ± 13.5	120.5 ± 13.2
Met-S (%)	50%	16% **
IFG and/or DM (%)	71.4%	16% **
Dyslipidemia (%)	45.2%	24% *
HT (%)	59.5%	32% *

**Table 2:** Pituitary hormone levels in subjects with and without Ni allergy. Abbreviations: SD, Standard Deviation; Ni, Nickel; GH, Growth Hormone; IGF-1, Insulin-like Growth Factor 1; TSH, thyroid stimulating hormone; FT4, Free Thyroxine; PRL, Prolactin; ACTH, Adrenocorticotropic hormone. * *p* < 0.05. ** *p* < 0.01. ^ *p* < 0.1.

Hormonal Status	Ni Allergy Confirmed (*n* = 42)	Ni Allergy Excluded (*n* = 25)
	Mean ± SD	Mean ± SD
GH (ng/mL)	0.27 ± 0.21	0.34 ± 0.31
IGF-1 (ng/mL)	128.3 ± 42.4	177.3 ± 78 *
TSH (µUI/mL)	2.2 ± 1.3	2.2 ± 1.2
FT4 (ng/dL)	1.2 ± 0.17	1.2 ± 0.18
PRL (ng/mL)	12.8 ± 6.2	13.7 ± 6.8
ACTH (pg/mL)	20.9 ± 11.7	32.6 ± 12.8 **
Cortisol (nmol/L)	130.2 ± 54.9	154.8 ± 47.8 ^

**Table 3:** Differences in pituitary morphological parameters between Ni allergic and non-Ni allergic patients in whole population and in the subgroup of patients without empty sella (ES). Abbreviations: SD, Standard Deviation; Ni, Nickel; ES, Empty Sella; T1W PTM intensity, T1-weighted pituitary to muscular mean signal intensity; T2W PTM intensity, T2-weighted pituitary to muscular mean signal intensity; NS, nonsignificant.

Pituitary Morphological Parameters	Ni Allergy Confirmed	Ni AllergyExcluded	*p*-Value
	Mean ± SD	Mean ± SD	
*Whole population (n)*	42	25	
Pituitary Volume (mm^2^)	389.5 ± 141.1	482.2 ± 124.9	0.004
Pituitary Height (mm)	4.3 ± 1.5	5.3 ± 1.9	0.02
Pituitary Width (mm)	14.6 ± 2.1	14.8 ± 2.2	NS
Pituitary Length (mm)	12.7 ± 1.9	12.6 ± 1.5	NS
Pituitary Stalk Thickness (mm)	2.44 ± 0.66	2.46 ± 0.71	NS
T1W PTM intensity	1.2 ± 0.16	1.40 ± 0.27	NS
T2W PTM intensity	4.9 ± 1.7	3.6 ± 1.18	0.005
*Patients with ES diagnosis excluded (n)*	23	18	
Pituitary Volume (mm^2^)	405.5 ± 151.8	500.5 ± 125.8	0.03
Pituitary Height (mm)	4.8 ± 1.6	5.7 ± 1.9	NS
Pituitary Width (mm)	14.3 ± 2.2	14.6 ± 1.9	NS
Pituitary Length (mm)	11.8 ± 1.5	12.1 ± 1.2	NS
Pituitary Stalk Thickness (mm)	2.60 ± 0.53	2.60 ± 0.69	NS
T1W PTM intensity	1.2 ± 0.16	1.38 ± 0.26	NS
T2W PTM intensity	4.9 ± 1.5	3.4 ± 1.3	0.02

**Table 4:** Bivariate correlations between Ni allergy, BMI, GH-IGF1 axis, pituitary morphology and inflammatory status parameters: correlations are expressed as Pearson correlation coefficients. Abbreviation: Ni A, Nickel Allergy; BMI, Body Mass Index; GH, Growth Hormone; IGF1, Insulin-like growth factor 1; GH peak, Growth Hormone peak upon GH Releasing Hormone (GHRH) plus Arginine dynamic test; PV, Pituitary Volume; T1W PTM intensity, T1-weighted pituitary to muscular mean signal intensity; T2W PTM intensity, T2-weighted pituitary to muscular mean signal intensity; WBC, White Blood Cells; L, Lymphocytes; N, Neutrophiles; CRP, C-Reactive Protein; ESR, Erythrocyte sedimentation rate. significant correlations are highlighted in bold. *** *p* < 0.001; ** *p* < 0.01; * *p* < 0.05 level.

	Ni A	BMI	GH	IGF1	GH Peak	PV	T1WPTM	T2WPTM	WBC	L(n)	N(n)	CRP	ESR	Ferritin
**Ni A**	1													
**BMI**	−0.01	1												
**GH**	−0.15	−0.12	1											
**IGF1**	−0.35 **	0.09	−0.01	1										
**GH peak**	−0.22	−0.15	0.05	0.24	1									
**PV**	−0.23 **	0.19	−0.04	0.21	0.08	1								
**T1WPTM**	0.13	0.01	0.04	0.16	−0.08	−0.2	1							
**T2WPTM**	0.39 **	0.14	0.04	0.23	0.06	−0.15	0.33 *	1						
**WBC**	−0.01	−0.03	−0.34 *	0.25 *	0.02	−0.09	−0.02	−0.04	1					
**L (n)**	0.04	0.13	−0.31 *	0.37 **	0.17	0.01	0.07	−0.02	0.56 ***	1				
**N (n)**	−0.04	−0.09	−0.29 *	0.16	−0.1	−0.09	−0.05	−0.06	0.94 ***	0.26 *	1			
**CRP**	0.09	−0.03	−0.29	−0.09	−0.26	−0.13	0.02	−0.16	0.32 *	0.12	0.32 *	1		
**ESR**	−0.14	−0.20	−0.13	0.09	−0.01	−0.05	−0.03	−0.19	0.28 *	0.13	0.28 *	0.44 **	1	
**Ferritin**	−0.14	−0.06	0.12	−0.15	0.21	−0.17	0.03	0.03	−0.01	0.18	−0.09	−0.07	0.12	1

**Table 5:** Partial correlations between Ni allergy, GH-IGF1 axis, pituitary morphology and inflammatory status parameters after BMI adjustment: correlations are expressed as Pearson correlation coefficients. Abbreviation: Ni A, Nickel Allergy; BMI, Body Mass Index; GH, Growth Hormone; IGF1, Insulin-like growth factor 1; GH peak, Growth Hormone peak upon GH Releasing Hormone (GHRH) plus Arginine dynamic test; PV, Pituitary Volume; T1W PTM intensity, T1-weighted pituitary to muscular mean signal intensity; T2W PTM intensity, T2-weighted pituitary to muscular mean signal intensity; WBC, White Blood Cells; L, Lymphocytes; N, Neutrophiles; CRP, C Reactive Protein; ESR, Erythrocyte sedimentation rate. significant correlations are highlighted in bold *** *p* < 0.001; * *p* < 0.05 level; ^ *p* < 0.1.

*6*BMI−Adj.	Ni A	GH	IGF1	GH Peak	PV	T1WPTM	T2WPTM	WBC	L(n)	N(n)	CRP	ESR	Ferritin
**Ni A**	1												
**GH**	−0.57	1											
**IGF1**	**−0.55 ^**	0.48	1										
**GH peak**	−0.37	0.68 *	0.47	1									
**PV**	**−0.38**	0.33	−0.21	0.46	1								
**T1WPTM**	−0.01	0.37	−0.01	−0.12	−0.19	1							
**T2WPTM**	**−0.16**	0.30	0.49	0.37	0.22	−0.28 *	1						
**WBC**	0.59 *	−0.53 *	**−0.07**	−0.45	−0.41	0.13	−0.07	1					
**L (n)**	**0.53 ^**	−0.27 *	0.24	−0.37	−0.43	0.20	0.21	0.81 ***	1				
**N (n)**	**0.53 ^**	−0.56 *	−0.14	−0.47	−0.28	0.05	−0.14	0.97 ***	0.56 *	1			
**CRP**	0.73 *	−0.40	−0.71 *	**−0.56 ^**	−0.28	0.32	−0.49	0.27	−0.04	0.32	1		
**ESR**	−0.24	−0.11	−0.18	0.36	0.63	−0.51	0.04	−0.16	−0.27	−0.05	−0.38	1	
**Ferritin**	−0.03	0.52	0.01	0.13	−0.26	0.47	−0.31	−0.14	0.02	−0.32	0.06	−0.26	1
